# Polymorphisms and plasma levels of IL-27: impact on genetic susceptibility and clinical outcome of bladder cancer

**DOI:** 10.1186/s12885-015-1459-7

**Published:** 2015-05-27

**Authors:** Bin Zhou, Peng Zhang, Tielong Tang, Hong Liao, Kui Zhang, Yan Pu, Peng Chen, Yaping Song, Lin Zhang

**Affiliations:** 1 Laboratory of Molecular Translational Medicine, West China Institute of Women and Children’s Health, Key Laboratory of Obstetric & Gynecologic and Pediatric Diseases and Birth Defects of Ministry of Education, West China Second University Hospital, Sichuan University, Chengdu, Sichuan P.R. China; 2 Department of Urology, West China Hospital, Sichuan University, Chengdu, Sichuan P.R. China; 3 Department of Urology, Affiliated Hospital of North Sichuan Medical College, Nanchong, Sichuan P.R. China; 4 Department of Urology, Institute of oncology, the Second People’s Hospital of Sichuan, Chengdu, P.R. China; 5 Department of Forensic Biology, West China School of Preclinical and Forensic Medicine, Sichuan University, Chengdu, Sichuan P.R. China

**Keywords:** Bladder cancer, IL-27, Polymorphisms, Plasma levels, Susceptibility, Prognosis

## Abstract

**Background:**

Interleukin-27 (IL-27) has been recognized as a pleiotropic cytokine with both pro- and anti-inflammatory properties. Few studies have investigated polymorphisms and serum/plasma levels of IL-27 in diseases including cancers. This study has analyzed the associations of *IL-27* gene polymorphisms, as well as plasma levels of IL-27, with susceptibility to bladder cancer and clinical outcome.

**Methods:**

Three hundred and thirty-two patients (nonmuscle-invasive bladder cancer (NMIBC)/muscle-invasive bladder cancer (MIBC): 176/156) included in a 60-month follow-up program and 499 controls were enrolled. Two single nucleotide polymorphisms (SNPs), rs153109 and rs17855750, were genotyped by polymerase chain reaction (PCR) -restriction fragment length polymorphism (RFLP) method. Plasma concentration of IL-27 was determined by ELISA in 124 patients (NMIBC/MIBC: 50/74) and 151 controls.

**Results:**

Significantly increased risk for bladder cancer was associated with AG/GG genotypes of rs153109 (*P* = 0.029). No GG genotype of rs17855750 was observed in controls, while 4 patients were found to be GG homozygotes, suggesting GG genotype may be associated with bladder cancer risk (*P* = 0.006). For bladder cancer patients, SNP rs17855750 was also associated with increased risk for MIBC. For MIBC patients, but not NMIBC, TG/GG genotypes of rs17855750 turned out to be a protective factor for overall survival (*P* = 0.035). Significantly reduced plasma levels of IL-27 were observed in both NMIBC and MIBC patients compared with controls (*P* < 0.0001).

**Conclusion:**

Our data suggest that polymorphisms and reduced plasma levels of IL-27 may predict the susceptibility to bladder cancer, and rs17855750 may be a useful marker to distinguish patients with high risk of death.

## Background

Bladder cancer is one of the most common cancers worldwide and the most frequent malignancy of the urinary tract [1]. In China, bladder cancer is the tenth most common cancer, accounting for 17,365 deaths in 2005 and mortality has steadily increased between 1991 and 2005 [2]. Transitional cell carcinoma of the urinary bladder represents more than 90 % of all bladder cancers, approximately 80 % of which are nonmuscle-invasive bladder cancer (NMIBC). The vast majority of cancer-specific deaths are due to muscle-invasive bladder cancer (MIBC), although only about 20 % of bladder cancer patients are diagnosed with MIBC. Tobacco smoking and occupational or environmental exposure to chemical carcinogens are the most and well-established risk factors for bladder cancer. However, only a few of the exposed individuals develop bladder cancer in their lifetime, suggesting that genetic factor may also play a crucial role in the pathogenesis of bladder cancer [3].

Cancer is a hyperproliferative disorder involving sustaining proliferative signaling, evading growth suppressors, resisting cell death, enabling replicative immortality, inducing angiogenesis, activating invasion and metastasis, reprogramming of energy metabolism, and evading immune destruction [4, 5]. Clinical and epidemiologic studies have suggested that inflammation can play a direct role in cancer [6, 7]. Inflammation, which orchestrates the tumor microenvironment, is involved in tumor initiation, promotion, and progression [8–10]. In bladder carcinogenesis, findings from numerous studies have suggested that inflammation is likely to have an important role [11]. C-reactive protein (CRP), which is an acute-phase reactant and a useful marker of systemic inflammation, has been shown to be a biomarker for bladder cancer [12–14].

Interleukin-27 (IL-27) is a heterodimeric cytokine composed of the Epstein-Barr virus-induced gene 3 (EBI3) and IL-27p28, which engages a receptor composed of gp130 and the IL-27Rα that activates Janus kinase (JAK)-signal transducer and activator of transcription (STAT) and mitogen activated protein kinase (MAPK) signaling [15, 16]. IL-27, which is a member of the IL-12 family of cytokines and chiefly produced by antigen-presenting cells (APC) such as dendritic cell (DCs) and macrophages, was initially described as a proinflammatory cytokine that promoted T helper (Th)1 responses [17]. Subsequent studies have confirmed an anti-inflammatory role for IL-27 in Th1, Th2, and Th17 responses, and it has also been shown that IL-27 can induce T cells to produce the anti-inflammatory cytokine IL-10 [18–20]. Of note, recent studies have revealed that IL-27 exerts potent antitumor effects against various tumor models via different mechanisms, including CD8^+^T cells, natural killer (NK) cells, antibody-dependent cell-mediated cytotoxicity (ADCC), antiangiogenesis, direct suppression of tumor growth, and inhibition of cychroxygenase-2 (COX-2) expression, depending on the characteristic of each tumor [21–25].

The associations between polymorphisms of *IL-27* gene, as well as serum/plasma levels of IL-27 and various human disorders including asthma, inflammatory bowel diseases, chronic obstructive pulmonary disease (COPD), colorectal cancer, esophageal cancer, and glioma have been studied [26–33]. Accordingly, the present study analyzed the influence of *IL-27* polymorphisms and plasma levels of IL-27 on the susceptibility to bladder cancer and prognosis of patients.

## Methods

### Study subjects

A hospital-based case–control study was conducted including 332 unrelated patients with transitional cell carcinoma of bladder between 2006 and 2010 derived from the West China Hospital of Sichuan University. The diagnosis of bladder cancer was confirmed in all cases by histological examination of tissue from resected specimens. Clinical and follow-up data were abstracted from patients’ medical records and by telephone calls. The control group consisted of 499 healthy subjects from a routine health survey in the same hospital. Table 1 summarized the baseline clinical characteristics of the patients and control groups. Those patients who had previous cancer, previous radiotherapy or chemotherapy, and metastasized cancer from other or unknown origins were excluded. Control subjects were genetically unrelated individuals and those with any personal or family history of bladder cancer or other serious disease were intentionally excluded. All subjects were Han population living in Sichuan province of southwest China. This study was approved by the ethics committee of West China Second University Hospital and all subjects gave written informed consent to participate.

**Table 1:** Characteristics of the study population

	NMIBC group	MIBC group	Controls
Sex			
Male	137 (77.8)	126 (80.8)	381 (76.4)
Female	39 (22.2)	30 (19.2)	118 (23.6)
Age at first diagnosis (mean ± SD)	61.96 ± 12.88	65.85 ± 10.98	64.51 ± 5.71
Smoking status			
Smokers	90 (51.1)	85 (54.5)	241 (48.3)
Non-smokers	86 (48.9)	71 (45.5)	258 (51.7)
Clinical stage			
Ta	10 (5.7)	-	-
T1	166 (94.3)	-	-
T2	-	91 (58.3)	-
T3a	-	36 (23.1)	-
T3b	-	18 (11.5)	-
T4	-	11 (7.1)	-
Tumor grade			
Low grade	119 (67.6)	25 (16.0)	-
High grade	57 (32.4)	131 (84.0)	-

### DNA extraction and genotyping

Two single nucleotide polymorphisms (SNPs), rs153109 (also known as −964 A/G) and rs17855750 (also known as 2905 T/G), were genotyped in the present study. Genomic DNA of each individual was extracted from 200 μl EDTA-anticoagulated peripheral blood samples by a DNA isolation kit from Bioteke (Peking, China) and the procedure was performed according to the manufacturer’s instructions. Genotyping was performed using the polymerase chain reaction (PCR) -restriction fragment length polymorphism (RFLP) method. Primers were established with the PIRA PCR designer (http://primer1.soton.ac.uk/primer2.html) [34]. In brief, the primer sequences were: F: 5′-CTGATCCTGACCTCACTCAACGC-3′ and R: 5′-CTGACTGGGACTGGGACTCAGC-3′ for rs153109. The primers used for amplification of rs17855750 were F: 5′-ATCTCGCCAGGAAGCTGCGC-3′ and R: 5′-CTGTTAGTGGGGGCCAGAAGGGA-3′.

DNA fragments containing the polymorphisms were amplified in a total volume of 25 μl, including 2.5 μl 10× PCR buffer, 1.5 mmol/L MgCl_2_, 0.15 mmol/L dNTPs, 0.5 μmol/L each primer, 100 ng of genomic DNA and 1 U of *Taq* DNA polymerase. The PCR conditions were 94 °C for 4 min, followed by 32 cycles of 30 s at 94 °C, 30 s at 64 °C for rs153109 and 66 °C for rs17855750, and 30 s at 72 °C, with a final elongation at 72 °C for 10 min. PCR products were digested overnight with specific restriction enzyme and the digested products were separated by a 6 % polyacrylamide gel and stained with 1.5 g/L argent nitrate: ***PaeR7I*** for rs153109, allele G is cuttable, yielding two fragments of 45 bp and 179 bp, allele A is uncuttable and the fragment is still 224 bp; and ***BstUI*** for rs17855750, allele G is cuttable, yielding two fragments of 19 bp and 101 bp, allele T is uncuttable and the fragment is still 120 bp. The genotypes were confirmed by the DNA sequencing analysis. About 10 % of the samples were randomly selected to perform the repeated assays and the results were 100 % concordant.

### Plasma IL-27 determination

For quantitative determination of IL-27, peripheral blood from 124 patients (NMIBC/MIBC: 50/74; male/female: 96/28) and 151 controls (male/female: 115/36) was collected into vaccutainer tubes containing EDTA-anticoagulant. Samples were centrifuged at 3000 × g for 10 min and plasma was collected and stored at −80 °C until use. Plasma levels of IL-27 were measured using commercially available enzyme-linked immunosorbent assay (ELISA) kits (USCNLIFE, Houston City, TX) according to the manufacturer’s instructions. Developed color reaction was measured as OD450 units on a multimode microplate reader (TECAN Infinite M200, Switzerland). The plasma concentration of IL-27 was determined using standard curve constructed with the kit’s standards over the range of 0–1000 pg/mL. The minimum detectable dose of IL-27 was typically less than 5.9 pg/mL.

### Statistical analyses

Data were analyzed using SPSS for Windows software package version 13.0 (SPSS Inc., Chicago, IL, USA). Genotype frequencies of these two SNPs were obtained by directed counting and Hardy-Weinberg equilibrium was evaluated by chi-square test. Odds ratio (OR) and respective 95 % confidence intervals (CI) were reported to evaluate the effects of any difference. Probability values of 0.05 or less were regarded as statistically significant, and all statistical tests were two sided. Genotypic association test in a case–control pattern assuming codominant, dominant, recessive, or overdominant genetic models was performed using SNPstats [35]. Allelic association was performed by chi-square test. The plasma IL-27 levels in patients with different *IL-27* SNPs genotype, and among NMIBC, MIBC and controls were compared using the non-parametric Kruskal-Wallis test. Tukey test was used for pairwise test. Mann Whitney test was used to compare plasma levels of IL-27 of subgroups (age at first diagnosis, sex, smoking status and tumor grade).

Kaplan-Meier plots and the log-rank test were used to evaluate the association between genotypes of *IL-27* SNPs, plasma IL-27 levels with patients’ outcome from the date of primary diagnosis until recurrence or death. Multivariate survival analysis for the influence of *IL-27* SNPs and plasma IL-27 levels on patients’ outcome was carried out by Cox regression analysis adjusted by the effect of age at first diagnosis, sex and smoking status. Hazard ratio (HR) and 95 % CI were calculated from the Cox regression model including all factors for multiple analysis.

## Results

### IL-27 SNPs and susceptibility to bladder cancer

These two SNPs of *IL-27*, rs153109 and rs17855750, were successfully genotyped in 332 patients with bladder cancer and 499 control subjects. Three genotypes of each SNP were identified and the genotypes were confirmed by the DNA sequencing analysis. All observed genotype frequencies in both patients and controls were in agreement with that expected under the Hardy-Weinberg equilibrium. Genotype distributions and allele frequencies of *IL-27* SNPs in patients and controls are shown in Table 2. Significant difference in genotype frequency distributions of rs153109 was observed between bladder cancer patients and controls (*P* = 0.029, OR = 1.37, 95 % CI = 1.03–1.82 for rs153109 in the dominant genetic model). For rs17855750, no GG genotype was observed in 499 controls, while there were 4 patients carrying GG in 322 patients, suggesting that rs17855750 may be associated with increased risk for bladder cancer in a recessive genetic model (*P* = 0.006). There were no statistically significant differences between patients and controls in terms of the allele frequency distribution of rs153109 and rs17855750.

**Table 2:** Distribution of SNPs in *IL-27* among patients and controls and their association with bladder cancer risk

		rs153109				rs17855750				
Model	Genotype	Controls	patients	OR (95 % CI)^a^	*P* value^a^	Genotype	Controls	patients	OR (95 % CI)^a^	*P* value^a^
		*N* = 499 (%)	*N* = 332 (%)				*N* = 499 (%)	*N* = 332 (%)		
Codominant	AA	229 (45.9)	127 (38.2 %)	1.00 (reference)	0.075	TT	421 (84.4)	275 (82.8 %)	1.00 (reference)	**0.022**
	AG	204 (40.9)	160 (48.2 %)	**1.41 (1.05**–**1.92)**		TG	78 (15.6)	53 (16.0 %)	0.97 (0.66–1.42)	
	GG	66 (13.2)	45 (13.6 %)	1.23 (0.79–1.89)		GG	0	4 (1.2 %)	0.00 (0.00-NA)	
Dominant	AA	229 (45.9)	127 (38.2 %)	1.00 (reference)	**0.029**	TT	421 (84.4)	275 (82.8 %)	1.00 (reference)	0.57
	AG/GG	270 (54.1)	205 (61.8 %)	**1.37 (1.03**–**1.82)**		TG/GG	78 (15.6)	57 (17.2 %)	0.90 (0.62–1.31)	
Recessive	AA/AG	433 (86.8)	287 (86.5 %)	1.00 (reference)	0.9	TT/TG	499 (100)	328 (98.8 %)	1.00 (reference)	**0.006**
	GG	66 (13.2)	45 (13.6 %)	1.03 (0.68–1.54)		GG	0 (0)	4 (1.2 %)	0.00 (0.00-NA)	
Overdominant	AA/GG	295 (59.1)	172 (51.8 %)	1.00 (reference)	**0.037**	TT/GG	421 (84.4)	279 (84.0 %)	1.00 (reference)	0.92
	AG	204 (40.9)	160 (48.2 %)	**1.35 (1.02**–**1.79)**		TG	78 (15.6)	53 (16.0 %)	0.98 (0.59–1.19)	
	Allele									
	A	662 (66.3)	414 (62.3)	1.19 (0.97–1.46)	0.096	T	920 (92.2)	603 (90.8)	1.20 (0.84–1.70)	0.317
	G	336 (33.7)	250 (37.7)			G	78 (7.8)	61 (9.2)		

*N* corresponds to the number of individuals

^a^Adjusted by age, sex and smoking status

Boldfaced values indicate a significant difference at the 5 % level

### IL-27 SNPs and patients’ characteristics

To further explore the effects of *IL-27* SNPs on bladder carcinogenesis, we conducted the stratified analyses. Genotypic distributions of rs153109 and rs17855750 between NMIBC and MIBC patients are shown in Table 3. No significant difference for the distribution of rs153109 between NMIBC and MIBC was observed. Compared to NMIBC, the TG/GG genotypes of rs17855750 were associated with an increased risk for MIBC (*P* = 0.042).

**Table 3:** Distribution of SNPs in *IL-27* among NMIBC and MIBC patients

		rs153109				rs17855750				
Model	Genotype	NMIBC	MIBC	OR (95 % CI)^a^	*P* value^a^	Genotype	NMIBC	MIBC	OR (95 % CI)^a^	*P* value^a^
		*N* = 176 (%)	*N* = 156 (%)				*N* = 176 (%)	*N* = 156 (%)		
Codominant	AA	66 (37.5 %)	61 (39.1 %)	1.00 (reference)	0.99	TT	149 (84.7 %)	126 (80.8 %)	1.00 (reference)	0.076
	AG	87 (49.4 %)	73 (46.8 %)	1.04 (0.60–1.81)		TG	26 (14.8 %)	27 (17.3 %)	0.53 (0.26–1.09)	
	GG	23 (13.1 %)	22 (14.1 %)	1.02 (0.46–2.27)		GG	1 (0.6 %)	3 (1.9 %)	0.15 (0.01–1.84)	
Dominant	AA	66 (37.5 %)	61 (39.1 %)	1.00 (reference)	0.88	TT	149 (84.7 %)	126 (80.8 %)	1.00 (reference)	**0.042**
	AG/GG	110 (62.5 %)	95 (60.9 %)	1.04 (0.62–1.75)		TG/GG	27 (15.3 %)	30 (19.2 %)	**2.04 (1.02**–**4.17)**	
Recessive	AA/AG	153 (86.9 %)	134 (85.9 %)	1.00 (reference)	0.99	TT/TG	175 (99.4 %)	153 (98.1 %)	1.00 (reference)	0.14
	GG	23 (13.1 %)	22 (14.1 %)	1.00 (0.48–2.09)		GG	1 (0.6 %)	3 (1.9 %)	0.17 (0.01–2.07)	
Overdominant	AA/GG	89 (50.6 %)	83 (53.2 %)	1.00 (reference)	0.88	TT/GG	150 (85.2 %)	129 (82.7 %)	1.00 (reference)	0.1
	AG	87 (49.4 %)	73 (46.8 %)	1.04 (0.63–1.73)		TG	26 (14.8 %)	27 (17.3 %)	0.55 (0.27–1.13)	
	Allele									
	A	219 (62.2)	195 (62.5)	0.99 (0.72–1.35)	0.94	T	324 (92.0)	279 (89.4)	1.37 (0.81–2.32)	0.28
	G	133 (37.8)	117 (37.5)			G	28 (8.0)	33 (10.6)		

*N* corresponds to the number of individuals

^a^Adjusted by age, sex and smoking status

Boldfaced values indicate a significant difference at the 5 % level

### Plasma IL-27 levels with susceptibility to bladder cancer and IL-27 genotype

**Fig. 1:**
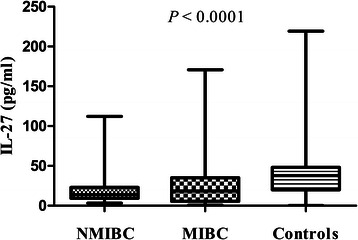
Plasma levels of IL-27 in NMIBC, MIBC patients and controls

As shown in Fig. 1 and Table 4, by analyzing the plasma IL-27 concentration of 124 bladder cancer patients and 151 controls, we found significant difference among NMIBC, MIBC and controls (*P* < 0.0001). Results of Tukey's Multiple Comparison Test showed IL-27 levels of NMIBC patients (21.16 ± 2.91 pg/ml) were significantly decreased compared with controls (38.21 ± 2.56 pg/ml). Although the IL-27 levels of MIBC patients (27.34 ± 5.72 pg/ml) were lower than that of controls, the difference was not statistically significant. There was no statistically significant difference for plasma IL-27 levels between NMIBC and MIBC patients.

**Table 4:** Relationship between plasma levels of IL-27 and patients’ characteristics

Variable analyzed		IL-27 (pg/ml)				
	Total, *N*	Mean	SD	SEM	Median	*P*
Case–control						
Controls	151	38.21	28.76	2.56	37.80	**< 0.0001**
NMIBC	74	21.16	21.92	2.91	13.91	
MIBC	50	27.34	32.87	5.72	18.41	
Age^a^						
≤65	88	26.49	32.23	4.8	16.33	0.94
>65	36	20.36	18.91	2.82	15.29	
Sex						
Male	96	24.13	27.14	3.22	16.33	0.43
Female	28	20.81	24.20	5.55	15.04	
Smoking status						
Smokers	73	22.86	25.80	3.42	16.33	0.55
Non-smokers	51	24.40	27.93	4.86	13.61	
Tumor grade						
Low grade	53	18.63	19.36	2.99	12.99	0.058
High grade	71	27.62	30.97	4.47	17.85	
rs153109						
AA	41	20.99	18.68	3.47	15.66	0.69
AG	61	26.07	31.37	4.44	15.69	
GG	22	17.80	18.45	5.56	13.51	
rs17855750						
TT	101	25.11	28.21	3.35	15.66	0.055
TG	20	17.13	17.64	4.05	10.93	
GG ^b^	3	-	-	-	-	

*N* corresponds to the number of individuals

^a^The threshold for age is based on median of bladder cancer patients

^b^Plasma IL-27 levels for all of these 3 samples were under the detection threshold for the present ELISA kits and their value was conservative determined as 5.9 pg/mL, which is the minimum detectable dose of IL-27 for the kits used in the present study

To study the association between genotype and phenotype, plasma IL-27 concentration in patients with respect to *IL-27* SNPs was investigated. As shown in Table 4, no significant association between plasma IL-27 levels and genotype of rs153109 was observed. Interestingly, plasma IL-27 levels for all of the 3 samples in rs17855750 GG group were under the detection threshold for the present ELISA kits and their value was conservative determined as 5.9 pg/mL, which is the minimum detectable dose of IL-27 for the kits used in the present study. Furthermore, the plasma IL-27 levels in patients with homozygous TT genotype were higher than that of heterozygous TG genotype, suggesting that genotype of rs17855750 may be associated with plasma IL-27 levels with an apparent gene-dose effect, although not statistically significant with a borderline *P* = 0.055.

No statistically significant association was found between plasma levels of IL-27 and patients’ characteristics including age, sex, or smoking status (Table 4). While the plasma IL-27 levels of patients with high grade (27.62 ± 4.47 pg/mL, n = 71) were higher than that of low grade (18.63 ± 2.99 pg/mL, n = 53), although not statistically significant (*P* = 0.058).

### IL-27 SNPs, plasma IL-27 levels and outcome

There were 332 bladder cancer patients included in this study. During the follow-up period, 51 patients (NMIBC: 13/176, MIBC: 38/156) had died and 281 patients alive (NMIBC: 163/176, MIBC: 118/156), and 97 patients (NMIBC: 50/176, MIBC: 47/156) had recurrence. The age of the patients who were dead was 69.22 ± 9.89 years compared with 62.80 ± 12.29 years in surviving patients (*P* < 0.001), and 65.03 ± 11.35 years in recurrent patients compared with 63.28 ± 12.47 years in non-recurrent patients (*P* = 0.23) . As it’s well known that patients with NMIBC have a better prognosis than patients with MIBC, survival analysis were conducted in these different populations independently.

**Fig. 2:**
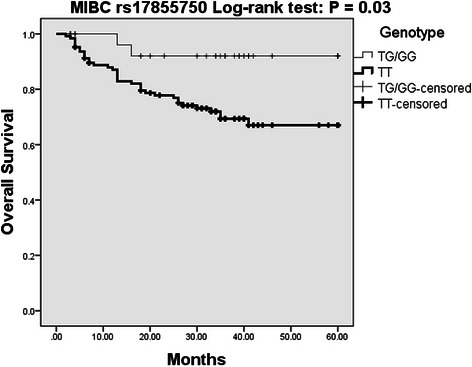
Kaplan-Meier overall survival curve for MIBC patients based on rs17855750 genotypes

**Fig. 3:**
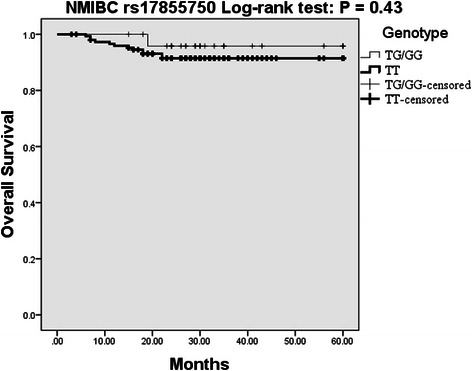
Kaplan-Meier overall survival curve for NMIBC patients based on rs17855750 genotypes

*IL-27* genotypes were subjected to multivariate survival analysis adjusted by age at first diagnosis, sex and smoking status (Table 5). No significant effect for rs153109 on overall survival or recurrence-free survival, and for rs17855750 on recurrence-free survival of both NMIBC and MIBC patients was observed. Interestingly, rs17855750 may be an independent protective factor for overall survival in patients with MIBC. In the dominant genetic model, univariate survival analysis indicated that MIBC patients with allele G (TG/GG genotype) had a significantly decreased risk for death than patients without allele G (TT genotype) (HR = 0.21, 95 % CI = 0.05–0.90, *P* = 0.035) (Fig. 2). However, there was no association between rs17855750 and overall survival of NMIBC patients (Fig. 3).

**Table 5:** Association between SNPs in IL-27 and patient’s survival

SNP/genotype				NMIBC						MIBC		
	Alive/dead, *N*	HR (95 % CI)^a^	*P*	Recurrence/Non-recurrence	HR (95 % CI)^a^	*P*	Alive/dead, *N*	HR (95 % CI)^a^	*P*	Recurrence/Non-recurrence	HR (95 % CI)^a^	*P*
rs153109												
AA	60/6			44/22			41/20			39/22		
AG	82/5			64/23			58/15			55/18		
GG	21/2			18/5			19/3			15/7		
Dominant		1.58 (0.53–4.72)	0.41		1.49 (0.85–2.60)	0.17		1.76 (0.93–3.33)	0.09		1.25 (0.70–2.23)	0.46
Recessive		1.02 (0.22–4.69)	0.98		0.68 (0.27–1.74)	0.42		0.42 (0.13–1.38)	0.15		0.91 (0.40–2.07)	0.82
Overdominant		0.63 (0.21–1.94)	0.42		0.80 (0.46–1.41)	0.44		0.81 (0.42–1.56)	0.53		0.84 (0.46–1.53)	0.56
rs17855750												
TT	137/12			105/44			90/36			87/39		
TG	25/1			20/6			26/1			20/7		
GG	1/0			1/0			2/1			2/1		
Dominant		0.39 (0.05–3.10)	0.37		0.59 (0.24–1.42)	0.24		**0.21 (0.05**–**0.90)**	**0.035**		0.81 (0.37–1.77)	0.60
Recessive		0 (0-NA)	0.99		0 (0-NA)	0.97		1.09 (0.14–8.45)	0.94		1.40 (0.18–10.76)	0.75
Overdominant		0.42 (0.05–3.35)	0.41		0.61 (0.25–1.49	0.28		**0.12 (0.02**–**0.88)**	**0.037**		0.76 (0.33–1.74)	0.52

*N* corresponds to the number of individuals

^a^Adjusted by age, sex and smoking status

Boldfaced values indicate a significant difference at the 5 % level

The association between plasma IL-27 levels and survival was analyzed in 124 patients (NMIBC: 74, MIBC: 50). During the follow-up period, 26 patients (NMIBC: 8/74, MIBC: 18/50) had died and 37 patients (NMIBC: 21/74, MIBC: 16/50) had recurrence. According to their respective median plasma IL-27 levels, 13.91 pg/ml for NMIBC and 18.41 pg/ml for MIBC, NMIBC and MIBC patients were divided into two subgroups, and the association of plasma IL-27 levels with survival was analyzed, respectively. We didn’t find any influence for the plasma IL-27 levels on patients’ survival.

## Discussion

In the present study, we have identified significant associations between *IL-27* SNPs and susceptibility to bladder cancer, patients’ characteristics, and overall survival of patients with MIBC. The association between different genotype of rs17855750 and plasma IL-27 levels, and significantly reduced plasma IL-27 levels compared to controls has also been observed.

IL-27, composed of the EBI3 and IL-27p28 subunits, is a member of the IL-12 family. EBI3 subunit was first identified from a subtractive hybridization screen of genes expressed in EBV transformed B cell lines in 1996 [36]. As a consequence of a computational approach to identify novel α-helical cytokines of the IL-6 family, the IL-27p28 subunit was recognized as the partner for EBI3 [17]. IL-27 could synergize with IL-12 to promote proliferation of naïve CD4^+^ T cells, but not memory CD4^+^ T cells, and the production of IFN-γ from NK cells and CD4^+^ T cells, suggesting that IL-27 function as a proinflammatory cytokine [17, 37]. Subsequent studies have revealed the pleiotropic properties of IL-27 that can limit or enhance ongoing immune responses depending on context.

It has been 10 years since the first report on the antitumor activity of IL-27 against a murine tumor model of colon carcinoma C26, which suggested that IL-27 has potent abilities to induce tumor-specific antitumor activity and protective immunity and that the antitumor activity is mediated mainly through CD8^+^ T cells, IFN-γ, and T-bet but not through STAT4 [24]. In the same year, results from TBJ murine neuroblastoma tumors also demonstrated that IL-27 has a potent ability to induce tumor-specific antitumor and protective immunity [38]. Since then, the last decade has seen the description of the signaling pathways engaged by IL-27, and an appreciation has emerged that IL-27 can modulate the intensity and duration of many classes of T cell responses [16]. IL-27 mediated antitumor mechanisms are complex, alone or in combination with other cytokines, IL-27 boosts antitumor immunity by contributing to the development of NK cells and cytotoxic T cells (CTLs), and by exerting potent antiangiogenic and antimetastatic activities [21].

The associations between polymorphisms of *IL-27* gene and human diseases, including cancers, have been widely studied [32, 33]. The SNP rs153109 has been reported to be associated with susceptibility to asthma and inflammatory bowel diseases in a Korean population, and with chronic obstructive pulmonary disease in a Chinese population, respectively [26–28]. Although no association between *IL-27* polymorphisms and immune thrombocytopenia, esophageal cancer, glioma, type 1 diabetes, or nasopharyngeal was observed in previous reports, our recent study suggested that *IL-27* gene polymorphisms may play important roles in the susceptibility to epithelial ovarian cancer [30, 31, 39–42]. The present study identified that *IL-27* gene is associated with susceptibility to bladder cancer. Furthermore, SNP rs17855750 turned out to be a protective factor for overall survival in patients with MIBC. For the first time, our data suggested that polymorphisms of *IL-27* gene may play important roles in the initiation, promotion, and progression of bladder cancer, especially in MIBC.

To date, few studies have reported the associations between serum/plasma IL-27 levels and human diseases. Although there was no association between *IL-27* gene polymorphisms and glioma, the serum IL-27 levels were decreased in glioma patients compared with controls. Moreover, the authors reported that *IL-12* gene 16974 A/C polymorphism may regulate the expression of the serum IL-12 and IL-27, but there were no significant associations of the *IL-27* gene polymorphisms with serum levels of IL-27 [31]. It has also been reported that serum/plasma levels of IL-27 decreased in epithelial ovarian cancer, while increased in cutaneous T-cell lymphoma, primary immune thrombocytopenia (ITP), and gastroesophageal cancer [41, 43–45]. Interestingly, although no significant association of rs153109 with plasma levels of IL-27 was observed, our present data indicated that genotype of rs17855750 may be associated with plasma IL-27 levels with an apparent gene-dose effect. Furthermore, plasma IL-27 levels for all of the 3 GG homozygous subjects which were identified only in the case group were under the detection threshold. The plasma levels of IL-27 among NMIBC, MIBC and controls were significantly different, especially IL-27 levels of NMIBC were significantly decreased compared with controls. While there was no difference between NMIBC and MIBC, or between MIBC and controls. We didn’t find any influence of plasma IL-27 levels on the survival of patients, the present data suggested that decreased plasma IL-27 levels may be associated with increased susceptibility to bladder cancer.

A growing body of evidence has validated the protective role of physiological levels of IL-27 against the development and progression of carcinogen- and transgene-driven neoplasms [22]. As it appears to have been associated with reduced toxicity, IL-27 might be advantageous over other cytokines in view of potential clinical applications [23]. No study of IL-27 on bladder cancer has been reported, but IL-27 has been reported to be effective in reducing tumor growth and can promote enhanced accumulation of effector cells in prostate tumors [46].

Taken together, the present study suggests that *IL-27* gene polymorphisms are associated with susceptibility to bladder cancer. The SNP rs17855750 may have a dual role as both a predictor of bladder cancer and a prognostic marker for patients’ survival. The decreased plasma levels of IL-27 compared to controls observed in our present study reinforces this predictive role as a marker of bladder cancer.

## Conclusions

In conclusion, although our findings should be interpreted cautiously due to the limited number of patients studied and sparse data, our present data lead us to speculate that the assessment of IL-27 gene polymorphisms and plasma levels may be useful for predicting susceptibility to bladder cancer and patients’ outcome. Further studies with a larger number of patients and IL-27 gene therapy in bladder cancer cells are warranted to confirm these results.
